# Biomass estimation equations for mesquite trees in the Americas

**DOI:** 10.7717/peerj.6782

**Published:** 2019-04-25

**Authors:** Jose Navar, Felipa de Jesus Rodriguez-Flores, Julio Rios-Saucedo

**Affiliations:** 1 Division de Estudios de Posgrado e Investigación, Tecnologico Nacional de Mexico/Instituto Tecnologico de Ciudad Victoria, Ciudad Victoria, Tamaulipas, Mexico; 2 Tecnologia Ambiental, Universidad Politecnica de Durango, Durango, Mexico; 3 Forestal, Instituto Nacional de Investigaciones Agricolas Forestales y Pecuarias, Durango, Mexico

**Keywords:** Regional aboveground biomass equation, Mesquite trees, Arid and semi-arid forests of the Americas

## Abstract

Mesquite trees are the preferred dendroenergy sources in arid and semi-arid forests. In spite of their relative importance, regional aboveground biomass (AGB) equations for mesquite trees are scarce in the scientific literature. For that reason, the aims of this study were to: (a) harvest trees and develop regional biomass equations; (b) contrast measured data with equations developed previously; and (c) test the applicability of the fitted equation for mesquite trees in the arid and semi-arid forests of the Americas. We harvested 206 new mesquite trees from arid and semi-arid forests in northern Mexico (Coahuila, Nuevo Leon, and Tamaulipas) in addition to using two other previously compiled data sets from Mexico (*N* = 304) to develop a regional equation. To test the validity of this equation, for biomass equations reported for the rest of the country, as well as for North and South American mesquite trees, we contrasted AGB measurements with predictions of fitted equations. Statistical analysis revealed the need for a single, regional, semi-empirical equation as together the three data sets represented the variability of the aboveground biomass of mesquite trees across northern Mexico, as well as mesquite trees in America’s arid and semiarid regions. Due to the large quantity of mesquite trees harvested for sampling and their variability, the regional biomass equation developed encompasses all other North and South American equations, and is representative of mesquite trees throughout the arid and semi-arid forests of the Americas.

## Introduction

Developing and applying allometric equations is the standard method for assessing tree, stand, regional, national, continental, and global aboveground biomass (Brown, 1997; Brown, Gillespie & Lugo, 1989; Chavé et al., 2005; Chavé et al., 2006). Biomass evaluations are prerequisites for assessing the stock and flux of several biogeochemical components including carbon and nitrogen (Brown, 1997; Chavé et al., 2005; Northup et al., 2005; McClaran, McMurtry & Archer, 2013; Chojnacky, Jenkins & Heath, 2014). Biomass estimation equations are also essential for evaluating belowground biomass as aboveground biomass (AGB) correlates well with this (Cairns & Brown, 1997; Mokany, Raison & Prokushkin, 2006). Complex hypotheses, such as optimal or allometric partitioning theories between tree communities, need to be tested using biomass compartment assessments (McCarthy & Enquist, 2007). In addition, AGB has the potential to become the primary global source of dendroenergy this century, and biomass assessments are vital for evaluating how much energy is stored in mesquite forests (McKendry, 2002; Berndes, Hogwijk & Den Broek, 2003).

AGB equations can be classified according to the parameter estimation method as empirical, semi-empirical, and theoretical (Návar, 2010). These models derive scaling coefficients using regression analysis (empirical), deterministic relations (theoretical), and a combination of both (semi-empirical). Compilations of AGB equations produced by Ter Mikaelian & Korzukhin (1997), Zianis & Mencuccini (2004) and Návar (2009) report that the conventional, most common empirical allometric equation is the logarithmic model where AGB is estimated as a log linear function of diameter at breast height (D) with two coefficients *a* and *b*. Preliminary findings showed that more theoretically based techniques did not provide the best estimation of AGB for mesquite trees (Návar, 2010). This suggests that more research is required to fit theoretically based equations and thus improve our understanding of biomass allometry of arid and semiarid trees. Other conventional, empirical, statistical equations should also be fit in order to preliminarily evaluate potential dendro-energy sources of mesquite trees for American arid and semiarid forests.

Mesquite trees are distributed throughout arid and semi-arid forests in the form of shrubs or low trees. They are the preferred tree species for dendroenergy in local households. Mesquite trees also represent a significant source of income for local populations as people trade dendroenergy, in the form of charcoal, in both local and international markets.

Regional aboveground biomass equations are therefore necessary for evaluating potential dendroenergy stocks, in the form of mesquite charcoal, in arid and semi-arid forests. Allometric equations reported in the literature are frequently local in nature and consistently fail to provide sound evaluations at the regional, national, or continental scales. This study therefore had the following objectives: (a) to harvest mesquite trees and to develop regional AGB equations; (b) to contrast AGB measurements with those equations developed previously; (c) to use a more comprehensive AGB data set for developing a regional equation and evaluate its appropriateness for assessing the AGB of mesquite trees in arid and semi-arid forests in Mexico and the Americas.

## Materials and Methods

### Study area

Mesquite trees are distributed throughout arid and semi-arid environments. In the Americas, these forests are found at latitudes of 15° to 40° both north and south of the equator. Regions with 8 to 10 dry months and which receive less than 750 mm per year of rainfall are classified as arid or semi-arid lands (García, 1987). In North America, it has been estimated that the Chihuahuan and Sonoran deserts, Great Plains, and dryland in most of Baja California comprise approximately 40–65% of the surface area of Mexico and the southern United States (Verbist et al., 2010). In South America, the Atacama Desert spanning Chile, Peru, and Bolivia; the Southern Patagonian forests of Argentina; and the dry forests of El Chaco in Paraguay and Argentina also have the climatic and vegetational characteristics of arid and semi-arid landscapes. Arid and semi-arid forests encompass ∼88 M ha (45%) of Mexico, and are primarily located in the northern portion of the country. Dryland forests are characterised by conspicuous shrubland and thorny savannah with isolated shrubs and trees.

Mesquite forests are found throughout these desert areas and are home to over 40 species of small leguminous trees (Rzedowski, 2006). *Prosopis glandulosa*, *Prosopis velutina*, *Prosopis juliflora*, *Prosopis laevigata*, and *Prosopis pallida* are common mesquite trees in North American dryland. *Prosopis flexuosa* and *Prosopis torquata*; *Prosopis caldenia*, *Prosopis hassleri*, *Prosopis pallida*, and *Prosopis nigra*; and *Prosopis tamarugo* range throughout the Argentinean, Paraguayan, and Chilean dryland of South America, respectively.

### Tree harvesting

We harvested 206 mesquite shrubs and low trees distributed across the northern Mexican Sates of Tamaulipas, Nuevo Leon, and Coahuila from 2006 to 2009. We felled trees and measured the basal diameter, Db, at c.a. 5 cm from the ground, diameter at breast height, D, top height, H. Harvested trees were dissected into their component parts. Then, we separated leaves and branches together and bole. Boles were logged to one meter in length for further commercial use. All leaves and branches, and logs were fresh weighted separately per tree. The total fresh weight of each component was obtained in the field using electronic balances and recorded to 1 g for material weighing less than 5 kg or to 10 g for heavier material. Samples of each component of each tree were fresh weighted and oven-dried (to constant weight at 70–80 °C). Dry weights were recorded to 0.1 g. Dry biomass was calculated by multiplying the dry to fresh weight ratios for each sample of each component by the fresh weight of the biomass component. Composite wood specific gravity was calculated for five trees at seven locations from 1-cm discs sawn from the main bole at diameter at breast height. Xilometers were used to measure disc volume by immersing each disc into the graduated tank. Disc dry weight was evaluated from the dry to fresh weight ratio described before. Total dry biomass, basal diameter, wood specific gravity for each individual tree for 206 trees made the data matrix for fitting previously developed allometric equations (Navar et al., 2013).

## Methods

### Fitted aboveground biomass equations

In this report, six AGB equations were fitted to two AGB data sets: two semi-empirical equations (West, Brown & Enquist, 1999; Návar, 2010); and four empirical (Conventional, Modified Conventional, Chojnacky, Jenkins & Heath, 2014; Jenkins et al., 2003). The harvested data set comprised 206 mesquite trees. These, together with information on 304 previously harvested mesquite trees from 1998 to 2004 (Navar et al., 2013), were also included in the second, full data set on a total of 510 mesquite trees harvested from across northern Mexico’s arid and semi-arid forests. Fitted allometric equations have previously been developed, tested and reported for arid and semi-arid shrubs (Navar et al., 2013). Firstly, this set of equations was fitted to the AGB data from the newly harvested trees and the goodness of fit statistics were evaluated. Secondly, the six AGB equations were fitted to the full data set (*N* = 510) to understand whether or not sample size influences scalar coefficients. Thirdly, a new set of scaling coefficients was evaluated for the full data set (*N* = 510) to determine the stability of these coefficients. Non-linear regression was used to evaluate the new set of scalar coefficients. The *C*-scalar coefficient in the West, Brown & Enquist (1999) and Návar (2010) equations must still be evaluated as well as any variation between tree species and between the trees in major forests may modify this coefficient. These equations are, therefore, currently semi-empirical in nature and the question remains of whether they need to be re-scaled for each tree species and for each location where mesquite trees are harvested.

1. The semi-empirical aboveground biomass equations used in this study:

1.1. West, Brown & Enquist (1999) model.

(1) \begin{eqnarray*}& & AGB= \left[ C{\rho }_{w} \right] {D}_{b}^{2.67}.\end{eqnarray*}

1.2. Constant *B*-scaling exponent model. Návar (2010) suggested that the mean scale coefficients of the conventional empirical model could be found in studies compiling empirical AGB equations. When diameter is measured at breast height, an average *B*-scaling value of 2.38 has been reported in meta-analysis studies of temperate and boreal tree species. The value of the *a*-scaling intercept is a function of the specific gravity of bole wood (Návar, 2010). With this assumption and the proposed statistical function between *a* vs *p*_*w*_, the suggested reduced model is

(2) \begin{eqnarray*}& & AGB= \left[ C{\rho }_{w} \right] {D}_{b}^{2.38}.\end{eqnarray*}

Where: *C* is a scaling constant, and *ρ*_*w*_ is the specific gravity of the entire AGB. In the West, Brown & Enquist (1999) and Návar (2010) equations, the exponent *B*_WBE_ is fixed to 8/3 = 2.67; *B*_NV_ = 2.38; and the specific gravity is the whole-tree specific gravity (a weighted average of wood, bark, branches and leaves).

2. Empirical equations.

2.1. Conventional equation fitted using non-linear regression. According to Navar et al. (2013), Eq. (3) describes the AGB of the arid and semi-arid shrubs and low trees of northeastern Mexico

(3) \begin{eqnarray*}& & AGB= \left[ \alpha \right] {D}_{b}^{{\beta }_{1}}.\end{eqnarray*}

Where: *α* and *B*_1_ are the scaling intercept (*B*_0_) and exponent (*B*_1_) of Eq. (3), respectively; both parameters are calculated by least square techniques using non-linear regression

2.2. Jenkins et al. (2003) equation. For North American woodlands that include mesquite trees, the equation developed by Jenkins et al. (2003) was applied to arid and semi-arid shrubs of northeastern Mexico with an intermediate level of precision. Replacing Db by D, the equation was originally reported as

(4) \begin{eqnarray*}& & AGB= \left[ 0.4891 \right] {D}_{b}^{1.7029}.\end{eqnarray*}

2.3. Chojnacky, Jenkins & Heath (2014) equation. For North American woodlands that include mesquite trees, the equation developed by Jenkins et al. (2003) was later modified by Chojnacky, Jenkins & Heath (2014) for arid and semi-arid North American woodlands that include tree species of the Families Fabacea and Rosacea (*Cercidium microphylum*, *Prosopis* spp., *Cercocarpus ledifolius*, and *C. montanus*). Replacing Db by D, the equation was originally reported as

(5) \begin{eqnarray*}& & Ln(AGB)=0.0536Ln({D}_{b}^{2.4109}).\end{eqnarray*}

Where: *Ln* = natural logarithm.

2.4. Modified conventional equation. The conventional equation requires the inclusion of the bole wood specific gravity in order to make it more useful in native forests with more than 2 tree species or in regional AGB evaluations of tree communities. This equation is mathematically described as

(6) \begin{eqnarray*}& & AGB= \left[ \alpha {\rho }_{w} \right] {D}_{b}^{{\beta }_{1}}.\end{eqnarray*}

Where: *α* and B_1_ are the statistical coefficients. Note that *α* ≈ C; and B_1_ ≈ 2.38 or 2.67.

Goodness-of-fit-statistics.

Wallach & Goffinet (1989) recommended models that are evaluated using two statistics: the standard error, *Sx*, Eq. (7); and the coefficient of determination or modelling efficiency, *r*^2^, Eq. (8).

(7) \begin{eqnarray*}& & {S}_{X}=\sqrt{ \frac{\sum _{i=1}^{n}{ \left( {y}_{i}-{\hat {y}}_{i} \right) }^{2}}{n-p-1} }\end{eqnarray*}

(8) \begin{eqnarray*}& & {r}^{2}=1- \frac{\sum _{i=1}^{n}{ \left( {y}_{i}-{\hat {y}}_{i} \right) }^{2}}{\sum _{i=1}^{n}{ \left( {y}_{i}-{\overline{y}}_{i} \right) }^{2}} .\end{eqnarray*}

Where: *y*_*i*_ is the measured aboveground biomass of tree *i*; ${\hat {y}}_{i}$ isthe estimated aboveground biomass of tree *i* by model *j*; $\overline{y}$ isthe mean measured aboveground biomass; and *p* is the number of statistical parameters or coefficients.

### Procedure

The results of the mesquite AGB equations reported for Mexico (Méndez et al., 2006; Mendez et al., 2012; Jimenez, 2013) were plotted within the measured AGB data set. In the same way, results of the reported mesquite AGB equations for other international locations: (i) Texas (Northup et al., 2005), (ii) Arizona (McClaran, McMurtry & Archer, 2013), and (iii) Argentina (Iglesias & Barchuk, 2010; Alvarez et al., 2011; Risio, Bravo & Bogino, 2012; Gaillard de Benitez et al., 2014; Chojnacky, Jenkins & Heath, 2014) were also plotted within the AGB measurements. The D of offsite AGB equations had to be transformed to Db, e.g., Gaillard de Benitez et al. (2014) and Chojnacky, Jenkins & Heath (2014), *Db* = *D*∕1.82. The plot was constructed using the basal diameters of the harvested trees to help understand if the offsite AGB equations fell within the measured data. This simple contrasting procedure provided compelling evidence of whether or not offsite allometric equations are sub-samples of the larger sample used to develop the regional equation. This procedure justifies the lack of other parameters such as *ρ*_*w*_ in the offsite that are required to run the recommended equation. Re-scaling coefficients of reported equations for the full data set (*N* = 510) was carried out in non-linear regression.

## Results

### Aboveground biomass data sets

**Figure 1:**
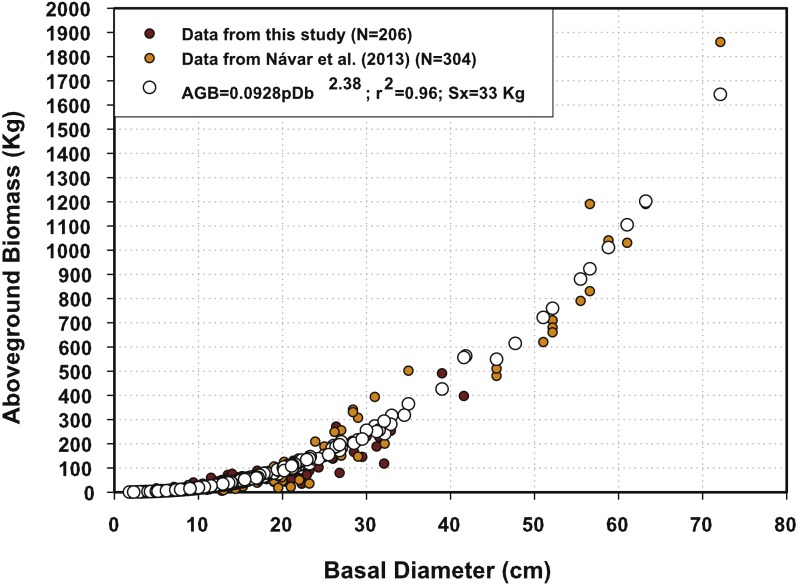
Predicted and measured aboveground biomass data sets using the semi-empirical model for northern Mexico’s mesquite trees.

Figure 1 depicts the two major AGB data sets. The data for this report gives similar AGB for Db < 40 cm, while the data collected from the rest of the country shows smaller AGB values for similar Db figures. The fact that the mesquite trees harvested may be from different species could explain the two clusters of AGB data. *Prosopis glandulosa* grows preferentially in the northern part of the country, whereas *Prosopis juliflora* and *Prosopis laevigata* are more commonly distributed in the rest of Mexico, more specifically at subtropical latitudes and in the dryland zone of the Mexican Altiplano. These differences are associated with shifts in the specific gravity values of the wood, as well as the canopy form. *P. glandulosa* has higher wood specific gravity values (0.70–0.80 g cm^−3^) and is a multi-stemmed shrub that eventually develops as a tree with the stems becoming branches. On the other hand, *P. juliflora* and *P. laevigata* have lower wood specific gravity values (0.60–0.70 g cm^−3^) and present most often a single, well-defined stem from early on in their developmental stages. In Mexico, *Prosopis* spp are under heavy browsing by different kinds of livestock modifying their structure and how biomass distributes along tree height. These major differences in plant traits and environmental stresses make the development of regional tree allometry more complex.

### Statistics

All six AGB equations with reported parameters showed a good fit (0.70 ≤ *r*^2^ ≤ 0.90) with the collected data set (*N* = 206). However, the Chojnacky, Jenkins & Heath (2014) and the semi-empirical equations more accurately estimated AGB, presenting a value of 0.85 ≤ *r*^2^ ≤ 0.90. The fit of the six equations with reported parameters improved when increasing the sample size from 206 to 510 data points, with the exceptions of the Jenkins et al. (2003) and Chojnacky, Jenkins & Heath (2014) equations (Table 1).

**Table 1:** Original fitted and re-scaled equations for assessing aboveground biomass of Mesquite trees across arid and semi-arid forests of the Americas.

	Fitted equations	
Author	Original	Re-scaled equations for mesquite trees
West, Brown & Enquist (1997)	$AGB= \left[ 0.0289 \right] {D}_{b}^{2.67}$	$AGB= \left[ 0.0295 \right] {D}_{b}^{2.67}$
Návar (2010)	$AGB= \left[ 0.0934{\rho }_{w} \right] {D}_{b}^{2.38}$	$AGB= \left[ 0.0928{\rho }_{w} \right] {D}_{b}^{2.38}$
Conventional	$AGB= \left[ 0.044 \right] {D}_{b}^{2.46}$	$AGB= \left[ 0.0877 \right] {D}_{b}^{2.30}$
Jenkins et al. (2003)	$AGB= \left[ 0.4891 \right] {D}_{b}^{1.7029}$	$AGB= \left[ 0.4891 \right] {D}_{b}^{1.7029}$
Chojnacky, Jenkins & Heath (2014)	$Ln(AGB)= \left[ -2.9255+2.426Ln{D}_{b} \right] $	$Ln(AGB)= \left[ -2.9255+2.426Ln{D}_{b} \right] $
Modified conventional	$AGB= \left[ 0.14{\rho }_{w} \right] {D}_{b}^{2.18}$	$AGB= \left[ 0.0645{\rho }_{w} \right] {D}_{b}^{2.47}$

**Notes.**

The equations originally reported by Navar et al. (2013) are for all shrub species of northern arid and semi-arid tree communities of Mexico.

The newly evaluated set of coefficients using the *N* = 510 data points clearly improved fit statistics for all equations with the exceptions of the Jenkins and Chojnacky equations. The semi-empirical models slightly modified the *C* coefficient and slightly improved the statistics, showing their prediction consistency across sample sizes and the stability of the *C*-scaling coefficient.

### Aboveground biomass equations

All applied AGB equations require the statistical coefficients to be re-scaled to improve the fit of the 510 AGB data points. The re-scaled AGB equations using the data from the 510 harvested mesquite trees are reported in Table 2.

**Table 2:** Goodness-of-fit statistics of six different equations fitted to aboveground biomass as a function of basal diameter for mesquite trees of northern arid and semi-arid forests of Mexico.

Author	Original equations		Original equations		New set of parameters	
	*N* = 206		*N* = 510		*N* = 510	
	*R*^2^	*Sx* (kg)	*R*^2^	*Sx* (kg)	*R*^2^	*Sx* (kg)
West, Brown & Enquist (1999)	0.89	24	0.96	35	0.96	35
Návar (2010)	0.85	28	0.96	33	0.96	33
Conventional	0.89	24	0.95	38	0.96	35
Jenkins et al. (2003)	0.78	34	0.70	91	0.70	91
Modified conventional	0.88	26	0.84	65	0.96	34
Chojnacky, Jenkins & Heath (2014)	0.90	23	0.95	37	0.90	53

For West, Brown & Enquist (1999) and Návar (2010), scaling coefficients for specific gravity were refit but theoretical exponents for basal diameter were not changed. In these equations the *C*-scaling coefficient must be modified slightly from 0.0289 and 0.0934 to 0.0295 and 0.0928 when re-scaling the original equations to adapt them for mesquite trees in dryland mesquite forests. The higher wood specific gravity values of mesquite trees compared to the other species in Tamaulipan thornscrub forests explain this increment in the scaling coefficients. However, these semi-empirical equations report consistent goodness-of-fit-statistics across different samples sizes (Table 1). In short, the AGB of mesquite trees is accurately evaluated, in a consistent way, using either of these two semi-empirical equations, highlighting their importance in local, regional, national, or continental AGB assessments.

The modified conventional allometric model also shifted the C and B-scaling coefficients when allowing the computer to statistically choose these. The new set of scaling coefficients was 0.14 and 2.18, differing slightly from the coefficients (0.0928 and 2.38) in the semi-empirical model of Návar (2010). When re-scaling the coefficients, the semi-empirical model of Návar (2010) recorded the highest goodness-of-fit- statistics for the data set containing all 510 trees, and the model shifted slightly the B-scaling coefficient for each data set. Then, when the model fits other AGB data sets it would probably bias insignificantly AGB evaluations and goodness-of-fit-statistics as well (Table 1). Therefore, the semi-empirical model makes good predictions of AGB for large regional mesquite AGB data sets, and provides consistent goodness-of-fit- statistics for small, local mesquite tree AGB data sets.

The semi-empirical AGB equations (West, Brown & Enquist, 1999; Návar, 2010) have the advantage of more consistently predicting AGB values across different sample sizes, from local (*N* = 206) to regional (*N* = 510) scale, and the statistics increase slightly with sample size. In order to further improve the goodness-of-fit-statistics, the *C*-scaling coefficient must be evaluated using the statistical relationship between re-scaled *a* versus *p*_*w*_ values for individual mesquite tree species. The slenderness factor (H/D) must also be incorporated into the *C*-scaling coefficient to improve AGB assessments. These issues should be addressed in future research.

### Contrasting model predictions with offsite equations

**Figure 2:**
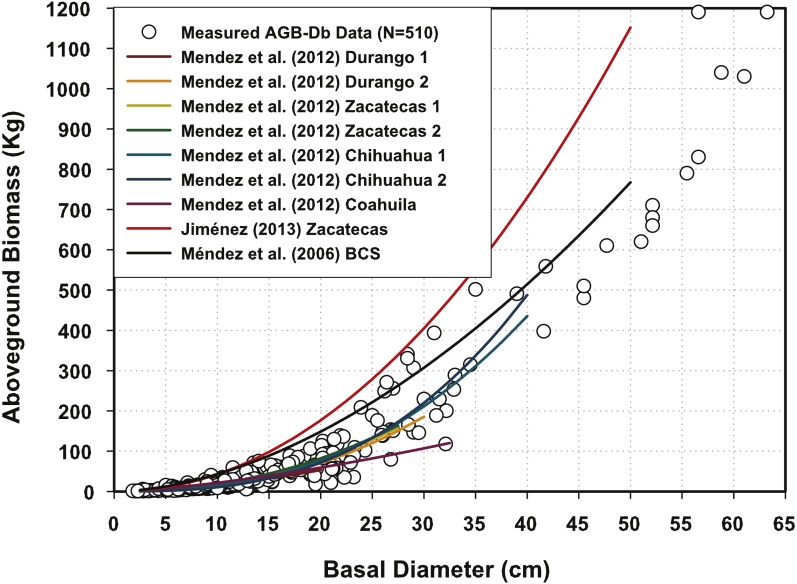
Aboveground biomass measurements plotted with predictions of other Mexican mesquite tree equations.

All the AGB equations previously reported for mesquite trees in Mexico (Méndez et al., 2006; Mendez et al., 2012; Jimenez, 2013) fall within the measured AGB data range (Fig. 2). The equation developed for Zacatecas, Mexico with Db > 30 cm, is the only exception. Harvesting trees with Db > 30 cm and re-scaling the coefficients would eventually determine whether the local or regional equations should be modified.

**Figure 3:**
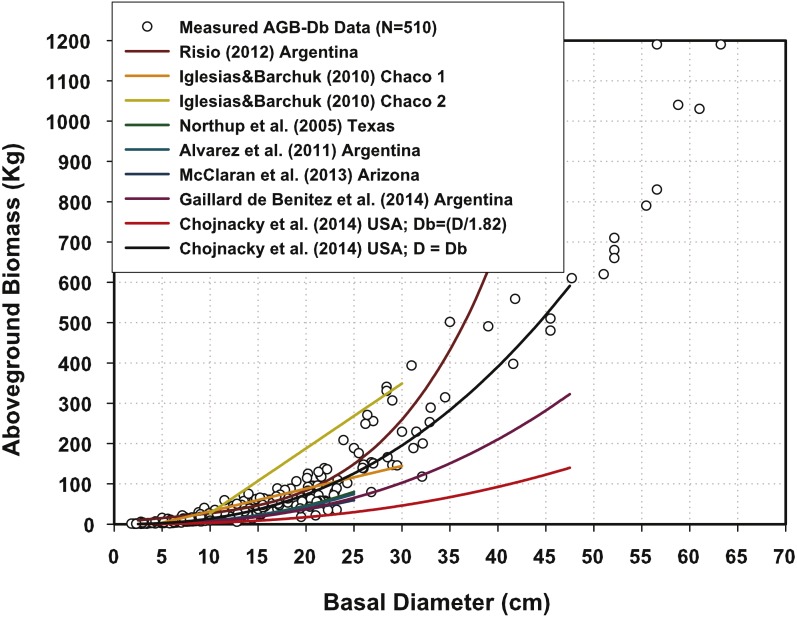
Aboveground biomass measurements plotted with predictions of offsite Mexican mesquite tree equations.

Figure 3 also reveals the fact that AGB data measured across northern Mexico consistently matches the AGB predictions for North American and South American mesquite trees presented by Northup et al. (2005), Iglesias & Barchuk (2010), Alvarez et al. (2011), Risio, Bravo & Bogino (2012), McClaran, McMurtry & Archer (2013) and Gaillard de Benitez et al. (2014), with the only exception of the AGB equation reported by Chojnacky, Jenkins & Heath (2014). The transformation of D into Db notoriously underestimates although the original equation matches perfectly measured AGB data. The question remains of what is the right factor to transform D into Db for mesquite trees across mesquite trees of the Americas. The equations developed for *P. glandulosa* in Texas (Northup et al., 2005); *P. velutina* in Arizona (McClaran, McMurtry & Archer, 2013); and *P. flexuosa* in Argentina (Alvarez et al., 2011) are within data but they lie at the lower limit of the AGB measurements for northern Mexico. The question remains of whether the local AGB equations necessitate the sampling of mesquite trees with Db > 30 cm in order to re-scale the coefficient *a* and exponent *B*. This should also be tackled in future research.

## Discussion

The findings of this research reveal: (i) a suite of allometric equations predict Mexican mesquite tree AGB well at the local, regional, national and continental scale; (ii) two semi-empirical models performed better than the empirical equations; (iii) the semi-empirical models predict compatible and consistent AGB assessments for dryland mesquite trees in the Americas.

The suite of allometric equations recommended for evaluating the AGB of mesquite trees in the dryland forests of the Americas are: Eqs. (1), (2), (3), (4) and (6). The parameters necessary for running these equations are: Db and *ρ*_*w*_ often measured and compiled in forest inventories. In the case that *ρ*_*w*_ is not available, Eqs. (3) and (4) still predict AGB well. Navar et al. (2013) recommended a computer equation (AGB = 0.075*Db*^2.65^*ah*^0.74^*p*_*w*^−0.277^_; *r*^2^ = 0.93; *Sx* = 19; *N* = 206; where: *ah* = *H*∕*Db*) that can be easily simplified by assuming an average wood specific gravity factor (e.g., 0.80 g cm^−3^ ± 0.07) and its power coefficient (−0.277), yielding an approximate average value of 1.06. In the same way, the slenderness factor (*ah* = *H*∕*Db*), which for mesquite trees of the northern Mexican Altiplano is a mean (confidence bound) value of 0.347 (±0.017), would yield an average value (0.347^0.74^) of 0.45 (±0.05). Multiplying 1.06 × 0.45 yields an approximate value of 0.48 and the computer equation converges to a simple conventional equation, AGB = 0.036*Db*^2.65^. However, this short version of the computer equation fails to provide compatible AGB evaluations as the full data set recorded poor goodness-of-fit-statistics (*r*^2^ = 0.48; *Sx* = 120 kg; *N* = 510). Wood density parameters have been compiled and reported by, for example, Chavé et al. (2006), Miles & Smith (2010) and Chojnacky, Jenkins & Heath (2014) that can be used to run Eqs. (1), (2) and (5).

The suite of allometric equations developed in this report can also be used to evaluate the local AGB of mesquite trees, although the Jenkins and Chojnacky equations Eqs. (4) and (6) would likely bias AGB assessments. For this reason, the semi-empirical Eqs. (1), (2) and (5) would provide more consistent and compatible AGB assessments. If the *ρ*_*w*_ is not available, Eq. (3) would still provide a good AGB assessment for local mesquite trees.

The fact the semi-empirical equations of West, Brown & Enquist (1999) and Návar (2010) provide consistent AGB evaluations across sample sizes with stable *C*-scaling coefficient values reveals the similar allometry principles and patterns that govern trees of different forests. In other words, the *β*-exponent coefficients are the same (*β* = 2.67 for Eq. (1) or *β* = 2.38 for Eq. (2)) for all forests, as stated previously by West, Brown & Enquist (1999). The statistically derived *C*-scaling coefficient for mesquite trees approximates to 0.0295 (Eq. (1)) or 0.0928 (Eq. (2)) depending on the equation employed. If using the Návar (2010) equation, the *C*-scaling coefficient is considerably less than the 0.2457 for North American temperate and boreal forests; and the 0.22 for Mexican temperate forests. This coefficient is related to stem taper and seems to be associated to the more exaggerated tapering of mesquite trees in contrast to that seen in pines or oaks of more mesic forests. A second simple explanation is that tree tapers more acutely when the diameter considered is Db and less acutely when from D. This should also be the object of further study.

Other statistical techniques for testing the equality of the AGB predictions for the different allometric equations are difficult to carry out as authors report different allometric equations and different techniques of estimating scaling coefficients. The preliminary statistics of the scaling and exponent coefficients (*a* and *B*) are the following: an average and median of the *a*-intercept (standard deviation, confidence bounds, *n*) of 0.27 and 0.127 (0.32, 0.14, 21); and *B*-slope of 2.17 and 2.19 (0.38, 0.16, 21). The *a*-intercept scalar coefficient does not distribute normal (*p* = 0.0002) unlike the *B*-slope scalar coefficient (*p* = 0.75). However, the *a*-intercept scaling coefficient distributed Weibull (*p* = 0.45), with the following density function coefficients: shape (*α*) = 0.642; scale (*β*) = 0.202; and location (ε) = 0.018. These parameters depict a distribution that is highly skewed to the right or an inverse *J*-distribution. The *β*-exponent coefficient also distributed Weibull (*p* = 0.77), with the following density function coefficients: shape (*α*) = 3.12; scale (*β*) = 1.183; and location (ε) = 1.119. These parameters represent a well-balanced distribution of the power exponent of AGB equations. These statistics also support the notion that mesquite biomass allometry is no different from the allometry of other, more mesic forests.

The group of mesquite trees includes more than 20 species. In North America, *P. glandulosa*, *P. laevigata*, *P. juliflora*, and *P. velutina* are common in arid and semi-arid forests. Each of these tree species has its own wood specific gravity value and its own canopy form; these are two major features involved in AGB allometry variability. Therefore, a single local empirical allometric equation would be insufficient to represent the regional variability of these features. This underlines the need for semi-empirical or fully theoretical equations in AGB evaluations.

## Conclusions

This study reports a suite of semi-empirical and empirical allometric equations for evaluating the AGB of mesquite trees at a local, regional, national, and continental scale. Of the fitted equations, the semi-empirical models provide the most compatible and consistent AGB assessments at these geographical scales for mesquite trees growing in arid and semi-arid woodlands and forests in Mexico and the Americas. They are therefore recommended for assessing dendroenergy components, such as charcoal from mesquite trees in arid and semi-arid zones.

##  Supplemental Information

10.7717/peerj.6782/supp-1 Dataset S1 Biomass Mesquite Data for Northern Mexico

## Additional Information and Declarations

### Data Availability

The following information was supplied regarding data availability: The raw data are available in Supplemental File.

## References

[ref-1] Alvarez JA, Villagra PE, Villalba R, Cony MA, Alberto M (2011). Wood productivity of Prosopis flexuosa DC woodlands in the central Monte: influence of population structure and tree-growth habit. Journal of Arid Environments.

[ref-2] Berndes G, Hogwijk M, Den Broek R (2003). The contribution of biomass in the future global energy supply. A review of 17 studies. Biomass and Bioenergy.

[ref-3] Brown S (1997). Estimating biomass and biomass change of tropical forests. Forest resources assessment publication. Forestry papers 134.

[ref-4] Brown S, Gillespie AJ, Lugo AE (1989). Biomass estimation methods for tropical forests with applications to forest inventory data. Forest Science.

[ref-5] Cairns MA, Brown S (1997). Root biomass allocation in the world’ s upland forests. Oecologia.

[ref-6] Chavé J, Andalo C, Brown S, Cairns MA, Chambers JQ, Eamus D, Folster H, Fromard F, Higuchi N, Kira T, Lescure J-P, Nelson BW, Ogawa H, Puig H, Riera B, Yamakura T (2005). Tree allometry and improved estimation of carbon stocks and balance in tropical forests. Oecologia.

[ref-7] Chavé J, Muller-Landau HC, Baker TR, Easdale TA, Steege H, Webb CO (2006). Regional and phylogenetic variation of wood density across 2,456 neotropical tree species. Ecological Applications.

[ref-8] Chojnacky DC, Jenkins JC, Heath LS (2014). Updated generalized biomass equations for North American tree species. Forestry.

[ref-9] Gaillard de Benitez C, Pece M, Juárez de Galíndez M, Maldonado A, Acosta M (2014). Modelaje de la biomasa aérea individual y otras relaciones dendrometricas de Prosopis nigra Gris. En la Provincia Santiago del Estero, Argentina. Quebracho.

[ref-10] García E (1987). Modificaciones al sistema de clasificación climática de Koppen (para adaptarlo a las condiciones de la República Mexicana).

[ref-11] Iglesias MR, Barchuk AH (2010). Estimación de biomasa aérea de seis leguminosas leñosas del Chaco árido (Argentina). Ecología Austral.

[ref-12] Jenkins JC, Chojnacky DC, Heath LS, Birdsey RA (2003). National-scale biomass estimators for United States trees species. Forest Science.

[ref-13] Jimenez E (2013). Modelos de predicción de volumen y biomasa de mezquite (Prosopis glandulosa Torr.) en Zaragoza, Coahuila. Revista Mexicana de Ciencias Forestales.

[ref-14] McCarthy MC, Enquist BJ (2007). Consistency between an allometric approach and optimal partitioning theory in global patterns of plant biomass allocation. Functional Ecology.

[ref-15] McClaran MP, McMurtry CR, Archer SR (2013). A tool for estimating impacts of woody encroachment in arid grasslands: allometric equations for biomass, carbon and nitrogen content in Prosopis velutina. Journal of Arid Environments.

[ref-16] McKendry P (2002). Energy production from biomass (part 1): overview of biomass. Bioresource Technology.

[ref-17] Méndez GJ, Santos MA, Nájera LJA, González OV (2006). Modelos para estimar volumen y biomasa de árboles individuales de Prosopis glandulosa, var. Torreyana en el Ejido Jesús González Ortega No 1, Mpio. De Mexicali, B.C. Recursos forestales, volumen 6, numero 2.

[ref-18] Mendez J, Turlan A, Ríos-Saucedo J, Najera A (2012). Ecuaciones alométricas para estimar biomasa aérea de Prosopis laevigata (Humb. & Bonpl. Ex Willd.) M.C. Johnst. Revista Mexicana de Ciencias Forestales.

[ref-19] Miles PD, Smith WD (2010). Specific gravity and other properties of wood and bark for 156 tree species found in North America. USDA forest service. Northern Rseaerch Station. Research note NRS-38.

[ref-20] Mokany K, Raison RJ, Prokushkin SA (2006). Critical analysis of root:shootratos in terrestrial biomes. Global Change Biology.

[ref-21] Návar J (2009). Biomass component equations for Latin American species and groups of species. Annals of Forest Science.

[ref-22] Návar J, Momba M, Bux F (2010). Measurement and assessment methods of forest aboveground biomass: a literature review and the challenges ahead. Biomass.

[ref-23] Navar J, Ríos-Saucedo J, Perez-Verdin G, Rodriguez-Flores FJ, Domínguez-Calleros PA (2013). Regional aboveground biomass equations for North American arid and semi arid forests. Journal of Arid Environments.

[ref-24] Northup BK, Zitzer SF, Archer S, McMurtry CR, Boutton TW (2005). Above-ground biomass and carbon and nitrogen content of woody species in a subtropical thornscrub parkland. Journal of Arid Environments.

[ref-25] Risio L, Bravo F, Bogino S (2012). Cuantificación de biomasa y carbono en bosques nativos de Prosopis caldenia (Burkhart) en la pampa semárida Argentina. http://uvadoc.uva.es/bitstream/10324/1593/1/TFM-L%2043pdf.

[ref-26] Rzedowski J (2006). Biodiversidad de México.

[ref-27] Ter Mikaelian MT, Korzukhin MD (1997). Biomass equations for sixty-five North American tree species. Forest Ecology and Management.

[ref-28] Verbist K, Santibañez F, Gabriels D, Soto G (2010). Atlas de zonas aridas de America Latina y el Caribe.

[ref-29] Wallach D, Goffinet B (1989). Mean squared error prediction as a criterion for evaluating and comparing system models. Ecological Modelling.

[ref-30] West GB, Brown JH, Enquist BJ (1997). A general model for the origin of allometric scaling laws in biology. Science.

[ref-31] West GB, Brown JH, Enquist BJ (1999). A general model for the structure and allometry of plant vascular system. Nature.

[ref-32] Zianis D, Mencuccini M (2004). On simplifying allometric analyses of forest biomass. Forest Ecology and Management.

